# The Netherlands Chlamydia cohort study (NECCST) protocol to assess the risk of late complications following *Chlamydia trachomatis* infection in women

**DOI:** 10.1186/s12879-017-2376-y

**Published:** 2017-04-11

**Authors:** B. M. Hoenderboom, A. A. M. van Oeffelen, B. H. B. van Benthem, J. E. A. M. van Bergen, N. H. T. M. Dukers-Muijrers, H. M. Götz, C. J. P. A. Hoebe, A. A. Hogewoning, F. R. M. van der Klis, D. van Baarle, J. A. Land, M. A. B. van der Sande, M. G. van Veen, F. de Vries, S. A. Morré, I. V. F. van den Broek

**Affiliations:** 1 Epidemiology and Surveillance Unit, Centre for Infectious Disease Control, National Institute for Public Health and the Environment, Bilthoven, The Netherlands; 2 Laboratory of Immunogenetics, Department Medical Microbiology and Infection Control, VU University Medical Center, Amsterdam, The Netherlands; 3 Department of General Practice, Division Clinical Methods and Public Health, Academic Medical Center, Amsterdam, the Netherlands; 4 STI AIDS Netherlands (SOA AIDS Nederland), Amsterdam, The Netherlands; 5 Department of Sexual Health, Infectious Diseases and Environmental Health, South Limburg Public Health Service (GGD South Limburg), Geleen, The Netherlands; 6 Department of Medical Microbiology, Care and Public Health Research Institute (CAPHRI), Maastricht University Medical Centre (MUMC+), Maastricht, The Netherlands; 7 Department Infectious Disease Control, Municipal Public Health Service Rotterdam-Rijnmond (GGD Rotterdam), Rotterdam, The Netherlands; 8 Department of Public Health, Erasmus MC—University Medical Center Rotterdam, Rotterdam, The Netherlands; 9 STI Outpatient Clinic, Public Health Service of Amsterdam (GGD Amsterdam), Amsterdam, The Netherlands; 10 Laboratory for Infectious Diseases and Perinatal Screening, Centre for Infectious Disease Control, National Institute of Public Health and the Environment, Bilthoven, The Netherlands; 11 Department Immune Mechanisms, Center for Infectious Disease control, National Institute for Public Health and the Environment, Bilthoven, The Netherlands; 12 Department of Obstetrics and Gynaecology, University Medical Center Groningen, Groningen, The Netherlands; 13 Julius Center for Health Sciences and Primary Care, University Medical Center Utrecht, Utrecht, The Netherlands; 14 Department of Clinical Pharmacology and Toxicology, Maastricht University Medical Centre (MUMC+), Maastricht, The Netherlands; 15 Institute for Public Health Genomics (IPHG), Department of Genetics and Cell Biology, Research School GROW (School for Oncology & Developmental Biology), Faculty of Health, Medicine & Life Sciences, University of Maastricht, Maastricht, The Netherlands

**Keywords:** *Chlamydia trachomatis*, Pelvic inflammatory disease, Tubal factor subfertility, Ectopic pregnancy, Host genetic biomarkers, Serology, The Netherlands

## Abstract

**Background:**

*Chlamydia trachomatis* (CT), the most common bacterial sexually transmitted infection (STI) among young women, can result in serious sequelae. Although the course of infection is often asymptomatic, CT may cause pelvic inflammatory disease (PID), leading to severe complications, such as prolonged time to pregnancy, ectopic pregnancy, and tubal factor subfertility. The risk of and risk factors for complications following CT-infection have not been assessed in a long-term prospective cohort study, the preferred design to define infections and complications adequately.

**Methods:**

In the Netherlands Chlamydia Cohort Study (NECCST), a cohort of women of reproductive age with and without a history of CT-infection is followed over a minimum of ten years to investigate (CT-related) reproductive tract complications. This study is a follow-up of the Chlamydia Screening Implementation (CSI) study, executed between 2008 and 2011 in the Netherlands. For NECCST, female CSI participants who consented to be approached for follow-up studies (*n* = 14,685) are invited, and prospectively followed until 2022. Four data collection moments are foreseen every two consecutive years. Questionnaire data and blood samples for CT-Immunoglobulin G (IgG) measurement are obtained as well as host DNA to determine specific genetic biomarkers related to susceptibility and severity of infection. CT-history will be based on CSI test outcomes, self-reported infections and CT-IgG presence. Information on (time to) pregnancies and the potential long-term complications (i.e. PID, ectopic pregnancy and (tubal factor) subfertility), will be acquired by questionnaires. Reported subfertility will be verified in medical registers. Occurrence of these late complications and prolonged time to pregnancy, as a proxy for reduced fertility due to a previous CT-infection, or other risk factors, will be investigated using longitudinal statistical procedures.

**Discussion:**

In the proposed study, the occurrence of late complications following CT-infection and its risk factors will be assessed. Ultimately, provided reliable risk factors and/or markers can be identified for such late complications. This will contribute to the development of a prognostic tool to estimate the risk of CT-related complications at an early time point, enabling targeted prevention and care towards women at risk for late complications.

**Trial registration:**

Dutch Trial Register NTR-5597. Retrospectively registered 14 February 2016.

## Background

*Chlamydia trachomatis* (CT) is the most commonly reported bacterial sexually transmitted infection (STI) in the Netherlands [1]. In contrast to most other STIs, CT is prevalent in a large segment of the population [2]. In the Netherlands, the reporting rate of CT-infections has steadily increased from 2.7/1000 persons in 2010 to 3.1/1000 in 2014, based on data from STI clinics and general practitioners (GPs). This is mainly due to increased testing rates in high-risk groups [1]. Enhanced testing in the Netherlands - as performed in the population-based Chlamydia Screening Implementation (CSI) study with annual screening between 2008 and 2011 - did not demonstrate a (cost-) effective reduction of CT prevalence, related to the relatively low and declining participation rates in the trial [3–5]. Observational studies in other European countries and a large CT screening pilot via general practitioners in Australia, showed similar results [6, 7]. In addition, in countries that have active screening policies (e.g. UK and USA) the number of CT-diagnoses in the population targeted for routine annual screening did not decline [6, 8]. New strategies for CT control are urgently needed.

Women bear a disproportional burden of CT-infections [9]. In the Netherlands, prevalence of CT around 2010 was estimated at 2.9% among 16–25 year-old women [5]. Since CT-infections in women have an asymptomatic course in up to 70% of the cases, most of these women will remain untreated [10, 11]. Meanwhile the CT-infection can ascend to the upper genital tract, resulting in pelvic inflammatory disease (PID), potentially causing tubal damage. Tubal damage in its turn may lead to ectopic pregnancy and tubal factor subfertility [12]. These complications only become apparent when women try to become pregnant, often several years after an initial CT-infection that may have gone unnoticed. A prior CT-infection may also prolong time to pregnancy in women without any visible tubal pathology, due to damage to the tubal mucosa (compromising embryo transport) or unfavourable effects in the endometrium (affecting implantation) [13].

The proportion of women experiencing CT-complications is largely unknown due to the asymptomatic nature of the infection, delayed awareness of the actual pathology, and the long follow-up period needed before complications become apparent [14]. Current risk estimates range widely; the estimated risk of PID following a CT-infection ranges between 0.5% and 72% depending on the study population and the definitions used [15–20]. Subfertility as a result of prior CT-infection is estimated to occur in 0.1–6% of women infected [15, 21], and 0–1% of women with a prior infection may develop an ectopic pregnancy [22, 23]. In a large retrospective population-based cohort of 500,000 women aged 15–44 years in Denmark, the risk of PID, ectopic pregnancy and tubal factor subfertility was found to be at least 30% higher in women who had tested CT-positive in the past compared to women who had only negative tests. In addition, repeated diagnoses of CT-infections increased the risk of PID by 22% [24]. Recent estimates by Price et al. in the UK, based on results of major studies and study designs, suggest that 20% of PIDs, 5% of ectopic pregnancies, and 30% of tubal factor subfertility cases are attributable to CT women aged 16–44 years [25].

Rather than attempting to trace and treat all CT-infections, it might be more effective to pursue secondary prevention in women at higher risk for complications. Host genetic biomarkers are considered to play a role in the development of complications in the reproductive tract after CT-infection [26]. In Gambian twin pairs, Bailey et al. found that 40% of the host response to trachoma (i.e. a tropical eye infection caused by CT serovar A or B) is linked to host genetic characteristics [27]. The scarring of the eye and the scarring of the tubes have a remarkable immunogenetic resemblance [26], and therefore we hypothesize a similar effect of host genetics in CT-related trachoma and tubal pathology. Subsequently, the presence of Single Nucleotide Polymorphisms (SNPs) in genes, identified by candidate gene studies and Genome Wide Association Studies (GWAS), has already been shown to be related to the development of tubal pathology following CT-infection. For example, carriage of two or more SNPs in toll-like receptor (TLR)9, TLR4, cluster of differentiation (CD)14 and caspase recruitment domain (CARD)15/nucleotide oligomerisation domain (NOD)2 increased the risk of tubal pathology following CT-infection from 33% to 73%, though the increase was not statistically significant [28]. Furthermore, carriership of mannose binding lectin (MBL) Codon 54 allele B was higher among CT-positives with tubal pathology (OR 3.9, 95%CI 1.9–8.2) [29] than among CT-positive controls and carriage of the NOD1 + 32,656 GG insertion was more frequent in women with TFI compared to women without TFI (OR 2.3, 95%CI 1.1–4.7) [30].

A range of other (host) factors such as clinical symptoms, co-infections, re-infections and sexual risk behaviour may influence the development of complications in women with a previous CT-infection [24, 31–33]. Some factors related to the (severity of) infection or sexual behavioural are time-dependent. To precisely quantify the risk and predisposing risk factors of PID, ectopic pregnancy and tubal factor subfertility following a CT-infection, prospective studies are needed to provide vital data for programs to prevent CT-infection and complications [14]. Therefore, the NEtherlands Chlamydia Cohort STudy (NECCST) was initiated. The strength of the study is its prospective design because this enables to collect, at regular time intervals, questionnaire-data with minimum (recall) bias, facilitates more extensive time analysis and allows to directly asses the risk of CT-related complications. The final aim will be to identify women most at risk for developing complications, in order to introduce targeted preventive measures and strategies to prevent CT (re-)infections in women at high risk for complications. Here we describe the NECCST cohort study design.

## Methods

### Study aim

With NECCST, we aim to gain more insight in late complications of CT-infections in women, and to identify women most at risk to develop CT-related complications. In this cohort study, the following aims will be addressed:

Primary objective:

- To quantify the incidence of PID, ectopic pregnancy and tubal factor subfertility and compare time to pregnancy in women with and without a previous CT-infection, in order to estimate the inherent risk of these outcomes by a previous CT-infection.

Secondary objectives:

- To explore which combination of host genetic biomarkers are able to distinguish women with a high risk of developing CT-related complications from women with a low risk of developing these complications.
- To determine demographic, behavioural, serological and infection-related factors that are associated with reproductive tract complications due to a preceding CT-infection.

## Design

NECCST is a long-term (10–14 years) cohort study, of women of reproductive age who previously participated in the CSI study.

### Setting

The starting point of the NECCST cohort is retrospective, at entry in the CSI study conducted between 2008 and 2011 in Amsterdam, Rotterdam and South Limburg (the Netherlands). In CSI, over 420,000 sexually active young adults (16–29 years old) who were registered in the municipal population registers of three areas (Amsterdam, Rotterdam and South-Limburg) were invited for annual CT-testing by home-based self-collection of a vaginal swab or urine sample. The samples were tested for a CT-infection using nucleic acid amplification tests (NAAT). In addition, participants completed questionnaires concerning demographic factors, sexual behaviour and previous STIs. Altogether, 80,000 people (19% of those invited) participated in CSI. Compared to non-responders, CSI participants were more often women, older (20–29 years), had a higher education, and were more often of a Dutch background. For the NECCST cohort, invitees were further selected from this group [4].

### Study population

All women who participated in at least one round of the CSI study (*n* = 58,818, 26% of those invited) and who gave informed consent to be approached for future STI-related research (14,685, 25%), are invited for participation in NECCST. Of the eligible women, 2371 (16%) had a positive CT-history (i.e. PCR positive result and/or self-reported CT-infection during CSI) and 12,314 (84%) had a negative CT-history according to CSI data (PCR negative and no self-reported previous CT-infection). These women are traced in the Dutch municipal registers and invited for participation in NECCST. Participants are between 20 and 38 years of age at the first data collection moment, and will be between 27 and 44 years of age by the end of NECCST. Women are censored when they have emigrated from the Netherlands or when contact details cannot be retrieved from the municipal registers for other reasons.

### Study procedures

NECCST covers a minimal individual follow-up period of 10 years and a maximum of 14 years, depending on inclusion in CSI, onwards. Participants will be contacted four times until 2022.

In 2015/2016, women were informed by regular mail and email (when available from CSI) about NECCST, and received an invitation letter and an information brochure. Via a web link in the invitation letter women could decline or accept participation. After one month a reminder is sent. After accepting, participating women were requested to complete an online informed consent form for participation in NECCST.

**Fig. 1:**
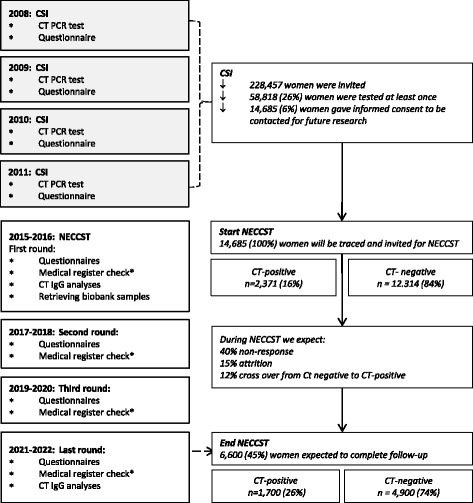
Flowchart of the study. *In case subfertility is reported, the participants’ medical files will be requested to verify the cause of subfertility. CSI = Chlamydia Screening Implementation. NECCST = Netherlands Chlamydia Cohort Study. CT = *Chlamydia trachomatis*. PCR = Polymerase chain reaction. IgG = Immunoglobulin G. CT-positive = at least one positive outcome, either a positive CSI CT Polymerase Chain Reaction (PCR) result, a self-reported CT-infection or CT IgG presence. CT-negative = Never tested positive during CSI, no self-reported CT-infection and no CT IgG presence

The first data collection moment includes an electronic questionnaire followed by a test kit for self-collection of blood by a finger-prick for CT IgG analyses. Host DNA is obtained by either using the stored CSI vaginal swabs or urine, or via additional newly self-collected buccal swabs [Copan FLOQSwabs™, Copan diagnostics, USA]. Every two years, an online questionnaire will be sent by email. In 2022, at the last data collection moment, a second test kit for self-collection of blood will be sent for CT IgG analyses (Fig. 1, study flowchart).

### Data collection during NECCST

#### Questionnaires

At the start of the NECCST follow-up period, women were asked to complete a baseline questionnaire. Thereafter, they receive a follow-up online questionnaire once every two years. The questionnaires inquire data about recent and past CT-infections and CT-related PID, time to pregnancy, ectopic pregnancy, and fertility problems. In addition, questionnaires will address demographic factors, age, ethnicity, educational level, sexual behaviour, other STIs, contraceptive use and health characteristics (e.g. smoking, weight changes, chronic pelvic pain (abdominal pain with a duration of 6 months or more) and previous abdominal surgery). Questionnaires are all electronic, sent with Formdesk [Formdesk, Wassenaar, The Netherlands], which creates a database automatically, linkable to other data sources (i.e. CSI-database), by individual participant number.

#### Medical register check

All women who report subfertility are asked additional informed consent to allow us to approach their GPs and/or fertility clinics to provide specified, detailed information regarding the cause of subfertility from the patient’s files in medical registers.

#### Chlamydia trachomatis serology

A test kit will be sent to participants to collect a capillary blood sample at home in a collection tube [BD Microtainer serum separator tube, Becton, Dickinson and Company, USA] and to return it to the laboratory in the accompanying packaging. All returned samples are immediately processed and blood collection tubes are centrifuged to collect serum. Serum samples are stored at −20 °C until an ELISA assay is performed. CT IgG antibodies are measured using a peptide based ELISA test [Medac CT IgG pELISA, Wedel, Germany] with minimal cross-reactivity and high throughput [34]. CT IgG antibody test results will be used as a marker for a previous CT-infection which remained unnoticed [35]. In addition, the seroconversion rate will be analysed in women with a previously self-reported or PCR confirmed CT-infection. An additional IgG antibody test will be performed in 2022, in order to determine new infections occurring during the study period and to gain insight in persistence of CT IgG levels over time.

#### Host genetic biomarkers

Host genetic biomarkers (SNPs), will be determined from host material obtained from vaginal swabs and urine samples collected during the CSI study and stored in a biobank. DNA will be extracted and host genetic biomarkers will be analysed using Kompetitive Allele Specific PCR (KASP) technology, utilizing a unique form of competitive allele-specific polymerase chain reaction (PCR). This enables accurate scoring of SNPs, inserts or deletions [36]. A selection of 50–100 SNPs will be tested. This SNP panel is based on previous research, that showed a potential association with chlamydia susceptibility and risk to develop complications after infection [26]. From participants whose previous CSI study sample is not available or of insufficient quality, a buccal swab sample is obtained at the start of NECCST in order to obtain DNA.

### Defining CT-history

As CT-infections often go unnoticed it was decided to define a positive CT-history based on one of the following three outcomes, either a self-reported CT-infection, positive PCR-test outcome in the CSI study or the presence of CT IgG antibodies in serum. Combining these three outcomes will reduce misclassification of CT-history.

### Power calculation

In total 14,685 women are invited of whom 2371 had a positive CT-history and 12,314 had a negative CT-history recorded in the CSI study. Assuming a high response rate of 60% (due to previous informed consent), a cross-over rate of 12% from negative to positive CT-history and an estimated loss to follow up of 25% until 2022, we expect to have 1700 women with a positive CT-history and 4900 with a negative CT-history participating in NECCST until the end of the study in 2022. Power calculations, based on the primary aim were performed using risk estimates from modelling and observational studies. In women with a positive CT-history the following risk estimates were taken into account: 10% for developing PID, 0.5% for ectopic pregnancy and 2% for tubal factor subfertility [15, 22, 23]. In women with a negative CT-history, the risk to develop these complications was estimated to be 0.5% for PID [37], and 0.05% for tubal factor subfertility [15]. For ectopic pregnancy, the expected risk ranges between 0 and 1% in CT negatives [22, 23]. Expected cumulative prevalence’s of these complicates were calculated based on age at the end of NECCST in 2022 (Table 1). The expected sample size of the study population, i.e. 1700 women with a positive CT-history and 4900 women with a negative CT-history, is sufficient to detect significant differences (*p* < 0.05) in risks of CT-related complications between women with and without a positive CT-history with a power of 85%–99%.

**Table 1:** Power calculation per outcome variable

	Expected cumulative prevalence in 2022		Number of samples needed for 80% power		Expected power with samples size *N* = 6600	
	CT pos. History	CT neg. History	CT pos. History	CT neg. History	CT pos. History (*n* = 1700)	CT neg. History (*n* = 4900)
PID	7.7%	1.1%	63	195	>99%	
Ectopic pregnancy	1.8%	0.9%	1415	4385	85%	
Tubal factor subfertility	1.3%	0.2%	401	1241	>99%	

Expected cumulative prevalence of PID, ectopic pregnancy and tubal factor subfertility was based on age distribution per outcome in the primary care database from the Netherlands Institute for Health Services Research (NIVEL – PCD) and the expected age distribution in NECCST in 2022 by CT status. Here from we derived the samples size, with 80% power, a significance level of 5% and a 1 to 3 exposed/unexposed ratio, per outcome in the CT-positive history and negative history group in 2022 (http://www.openepi.com). *CT Chlamydia trachomatis* infection, *PID* pelvic inflammatory disease

### Data analysis and statistics

Data from the CSI-study will be merged with data from NECCST. Data quality will be assessed, in particular the potential for bias due to non-response and the extent of missing data. Incidence rates, calculated as the number of new cases divided by the total person-time at risk, of the primary study endpoints PID, ectopic pregnancies and tubal factor subfertility will be compared between women with and without a positive CT-history. Person-time at risk is calculated from the time point a woman becomes sexually active (assessed from the CSI questionnaire) and ends at the time of an event. In case of no event, person-time at risk stops at the end of participation, migration out of the Netherlands or the end of the study period, whichever comes first.

Each of the primary study endpoints PID, ectopic pregnancy and tubal factor subfertility will be analysed for women with and without a positive CT-history using Kaplan-Meier plots and log-rank test. Cox proportional hazards regression analyses will be performed to calculated hazard ratios, in which CT-history is included as a time-dependent variable. Once a participant becomes CT-positive, the participant switches over to the CT-positive group. We will explore if time to infection for women with a positive CT-history based only on their CT IgG presence without further information on the time of infection, can be estimated using information from the group with both a positive IgG test result *and* a positive PCR test or self-reported CT-infection. To account for confounding and effect modification, co-factors such as age, educational level, host genetic biomarkers, demographics, behavioural and infection characteristics (i.e. as previous treatment and other STI’s), will be included in the model.

Similar analyses will be done to assess the factors related to time to pregnancy. For time to pregnancy the follow-up period is defined as the time in months between the moment the woman reports starting to try to conceive until the start of pregnancy, or (if not pregnant) the date of completion of the questionnaire.

Univariate and multivariate Cox regression analyses will also be applied to investigate covariates among women with a positive CT-history and the event of a PID, ectopic pregnancy or tubal factor subfertility. All factors that are significantly associated with the specific complication will be included in a multivariate Cox proportional hazards regression model. Using backward stepwise selection, factors that are not associated with the development of the complication anymore (with a threshold *p*-value of 0.1) will be removed. All other factors will be assigned significantly associated with the development of the complication. The following literature-based variables will be selected as a potential predictor: age, educational level, host genetic biomarkers, and demographic, behavioural and infection characteristics.

Sensitivity analyses will be performed by varying the definitions of a positive CT-history, i.e. only CSI-PCR positive outcomes compared to self-reported CT-infections and CT IgG positivity. We will also perform sensitivity analyses on variable definition of outcome variables, such as planned versus unplanned pregnancies and confirmed versus unconfirmed tubal factor subfertility.

## Discussion

In NECCST, the risk of PID, time to pregnancy, tubal factor subfertility and ectopic pregnancy after CT-infection will be determined in a cohort of women of reproductive age with an individual follow-up time of 10 to 14 years. NECCST will investigate the role of a wide range of host- and infection-specific factors in the development of CT-related complications. Insight in risk factors of CT-related complications may allow for a new strategy in prevention of the complications of CT. This could be an alternative approach in addition to current chlamydia control strategies, aiming to test and treat to prevent ongoing transmission. The ultimate goal will be to develop a prognostic tool to identify the group of women with an enhanced risk of complications after a CT-infection at an early time point in their life, when (secondary) preventive measures can be effectively applied. Women at high risk for complications could be specifically targeted for prevention of (re-)infection, e.g. by frequent follow-up as an optimal strategy for preventing long-term complications [24].

### Strengths

NECCST will be the first cohort study in which risks of and for late complications of CT are prospectively studied during a follow-up period of more than 10 years. A prospective study design allows for a clear temporal sequence of exposure and outcome and examination of multiple effects of a single exposure while avoiding selection bias at enrolment. The long-term follow-up is needed to examine the relation between exposure and outcome, as CT-infections are often acquired below the age of 20, fertility problems will only become apparent when a woman tries to become pregnant, which is often in her late twenties or thirties.

This cohort combines several data sources: historical data from the CSI, newly acquired questionnaire data at four time points during NECCST, medical register data, serological outcomes and host genetic biomarkers. By combining information from tests from the CSI study, self-reported CT-infections and CT IgG status, we expect to obtain a more complete picture of previous CT-infections than in most other studies. As we make use of serology, we will also be able to identify women who had a CT-infection which remained unnoticed and was therefore left untreated. These women can be compared to women who tested positive for CT and were treated accordingly. Comparing these groups may give us more insight into the IgG status and risk of CT-related complications between treated and untreated women and allow us to study the natural course of a CT-infection [14].

Host genetic biomarkers are nowadays implemented in prediction models and health care systems. Developing a set of specific genetic biomarkers associated with a high risk for CT-related complications can facilitate identifying the group of women most vulnerable for developing complications [26, 38]. With a ‘precision (or personalized) medicine approach’ a diagnostic tool on the basis of women’s genetic profile can be employed for selecting the group to be targeted for specific (cost)effective interventions such as repeated CT-testing, additional treatment and medical follow-up.

### Limitations

Although in our study CT-history will be based on PCR results from the CSI study, self-reported infections and CT IgG measurements, misclassifications may still occur. Women with an unnoticed infection may have a negative CT IgG antibody test, because an infection not always leads to CT IgG antibody production, or because the levels of antibodies have waned since the time of infection. The proportion of infected women who seroconvert and who remain persistently seropositive is not well established yet [35]. Sensitivity and specificity of CT IgG measurement of the assay we will use in NECCST (Medac pELISA) were reported to be 71.4% and 97.3% at the time of CT-infection, respectively [39]. However, within six months after infection, seropositivity was 66% and after six months or longer, seropositivity decreased to 38% [40], resulting in CT-positives possibly being misclassified as CT-negative. Because this could result in underestimation of the true association between CT-history and complications, we will perform sensitivity analyses in which only CSI-PCR positives will be classified as positive.

In our study, outcomes such as PID, tubal factor subfertility, ectopic pregnancy and time to pregnancy will be (initially) based on self-reporting, which may induce recall bias despite multiple questionnaire rounds every two years. The diagnosis of PID is imprecise and lacks a non-invasive accurate gold standard test [41]. Diagnostic bias could take place towards women with a positive CT-history [14]. This may result in a more pronounced underestimation of the risk on PID among CT-negatives than among CT-positives, potentially leading to an overestimation of the difference in PID risk between those groups. Therefore, in de questionnaire we specifically inquire about diagnosis by GP and hospital admissions for PID, and recall bias will be reduced. As a rough proxy for silent PID episodes, women are asked if they experienced chronic pelvic pain defined as unexplained abdominal pain with a duration of 6 months or more [33, 42]. A range of other causes besides PID can result in chronic pelvic pain as well. However, assuming the incidences of these other causes to be evenly distributed between CT-positives and CT-negatives, any differences in the occurrence of chronic pelvic pain may be considered the result of PID. This will increase power and cover both symptomatic and silent PIDs.

In participants with an asymptomatic CT-infection we will not be able to determine when the first CT-infection and tubal scarring that eventually leads to subfertility have occurred. We cannot rule out that in some cases tubal damage was already present before the CT-infection. But in general tubal scarring may be assumed to occur after infection.

Finally, selection bias may occur when women who have experienced reproductive tract problems and have had a CT-infection are more willing to participate in NECCST. However, we expect that based on the relatively young age at the inclusion in NECCST, a substantial proportion of participants will not yet have tried to become pregnant when giving their consent to participate. Nevertheless, sensitivity analyses will be performed to compare outcomes in participants who were aware of reproductive complications at the start of NECCST with outcomes in those who were not yet aware.

### In summary

In the future, instead of striving to detect and treat all CT-infections, chlamydia control strategies could focus on prevention of complications following a CT-infection. Long-term complications in women are the most important burden of CT-infections at population level. With this cohort study, we aim to contribute to better insight and further understanding of the factors involved in the development of late CT-complications, and to identify markers to understand which women are at risk of such complications. This should allow improved and targeted interventions to control adverse outcomes of CT infections.

## Abbreviations

CARD: Caspase recruitment domain
CD: Cluster of differentiation
CSI: Chlamydia screening implementation
CT: *Chlamydia trachomatis*
DNA: deoxyribonucleic acid
IgG: Immunoglobulin G
KASP: Kompetitive Allele Specific PCR
NAAT: Nucleic acid amplification test
NOD: Nucleotide oligomerisation domain
PCR: Polymerase chain reaction
PID: Pelvic inflammatory disease
SNP: Single nucleotide polymorphism
STI: Sexually transmitted infection
TLR: Toll-like receptor
